# Targeted Isolation of Anti-Trypanosomal Naphthofuran-Quinone Compounds from the Mangrove Plant *Avicennia lanata*

**DOI:** 10.3390/md18120661

**Published:** 2020-12-21

**Authors:** Noor Wini Mazlan, Carol Clements, RuAngelie Edrada-Ebel

**Affiliations:** 1 Strathclyde Institute of Pharmacy and Biomedical Sciences, University of Strathclyde, The John Arbuthnott Building, 161 Cathedral Street, Glasgow G4 0RE, UK; carol.clements@strath.ac.uk; 2 Faculty of Science and Marine Environment, Universiti Malaysia Terengganu, Kuala Nerus 21030, Terengganu, Malaysia; 3 Institute of Marine Biotechnology, Universiti Malaysia Terengganu, Kuala Nerus 21030, Terengganu, Malaysia

**Keywords:** mangrove, mass spectrometry, NMR, metabolic profiling, dereplication, multivariate analysis, high-throughput chromatographic

## Abstract

The discovery of new secondary metabolites from natural origins has become more challenging in natural products research. Different approaches have been applied to target the isolation of new bioactive metabolites from plant extracts. In this study, bioactive natural products were isolated from the crude organic extract of the mangrove plant *Avicennia lanata* collected from the east coast of Peninsular Malaysia in the Setiu Wetlands, Terengganu, using HRESI-LCMS-based metabolomics-guided isolation and fractionation. Isolation work on the crude extract *A. lanata* used high-throughput chromatographic techniques to give two new naphthofuranquinone derivatives, hydroxyavicenol C (**1**) and glycosemiquinone (**2**), along with the known compounds avicenol C (**3**), avicequinone C (**4**), glycoquinone (**5**), taraxerone (**6**), taraxerol (**7**), β-sitosterol (**8**) and stigmasterol (**9**). The elucidation and identification of the targeted bioactive compounds used 1D and 2D-NMR and mass spectrometry. Except for **6–9**, all isolated naphthoquinone compounds (**1–5**) from the mangrove plant *A. lanata* showed significant anti-trypanosomal activity on *Trypanosoma brucei brucei* with MIC values of 3.12–12.5 μM. Preliminary cytotoxicity screening against normal prostate cells (PNT2A) was also performed. All compounds exhibited low cytotoxicity, with compounds **3** and **4** showing moderate cytotoxicity of 78.3% and 68.6% of the control values at 100 μg/mL, respectively.

## 1. Introduction

Mangrove plants as well as their endophytic fungi exhibit unique chemical diversity from various classes of compounds with promising biological activities [1,2,3]. Avicennia is the only mangrove genus belonging to the Avicenniaceae family; it is the most abundant genus in mangrove ecosystems and is widely distributed on tropical and subtropical coastlines. Eight to ten species have been recorded worldwide [4]. *Avicennia lanata* (synonym: *A. rumphiana*), locally known in Malaysia as ‘*Api-api bulu*’, is found mainly in sandy or firm silt substrate of middle to higher intertidal zones [5]. It is native and common throughout much of Peninsular Malaysia, Philippines and New Guinea. This tree is identified by its furry underside leaves and fruit. The pelt (*‘bulu’* in Malay) on the leaves conserves water by trapping a layer of insulating air, thus reducing water loss through evaporation. The tree or shrub can grow up to 20 m tall; the bark is dark brown to black, warty or smooth, with pneumatophores 20–30 cm tall. The first phytochemical study on *Avicennia* sp. revealed lapachol, a naphthofuranquinone compound which has been isolated from Indian and West African *A. tomentosa* [6]. Later, various classes of chemical components have been isolated from the Avicennia genus, including naphthoquinones [7,8,9,10,11], iridoid glucosides [12,13,14,15,16,17], sterols [18,19], flavones [16,20], diterpenes [21] and triterpenes [18,19,22,23] from leaves, roots, twigs and stem bark.

Natural products research has an important role in the discovery of various biologically active substances of natural origin for potential new drugs. In natural product metabolomics research, the term ‘metabolite’ is usually referred to a group of small molecules [24,25]. These are classified into primary and secondary metabolites. Primary metabolites, which include amino acids, lipids and carbohydrates, refer to molecules that are required to support the growth function of an organism via normal metabolic process. Secondary metabolites, including polyphenols, alkaloids, terpenes, polyketides and hormones, are molecules related to signalling mechanisms for an organism’s defence and survival [26]. Some of these compounds possess potent activity in certain targeted biological tests, making them valuable in drug discovery and development. Human African trypanosomiasis is a neglected disease that requires international efforts for the development of new potential alternative drugs. Many of the affected rural populations have limited access to appropriate healthcare, and the production of anti-trypanosomal drugs is costly. Moreover, the available drugs used for the treatment of trypanosomiasis depend on the sub-species of the trypanosomes as well as the stage of the disease. The requirement for drugs that are able to cross the blood brain barrier to get into contact with the parasites is also a major challenge in drug design, since some drugs are difficult to administer, are toxic, and cause adverse drug reactions [27,28] The current situation necessitates the development of new, effective, cheap and safe remedies to combat the trypanosomiasis. Even now, there are no drugs of natural origin available commercially that can treat the disease. However, society has typically relied on traditional medicine from natural sources to heal a wide range of diseases [29,30,31] and indeed, previous studies on naphthofuranquinone metabolites which have been isolated from several plant sources have revealed promising anti-trypanosomal activity [32,33,34]. This has driven us to intensify our search for novel anti-trypanosomal agents from natural sources [35]. 

The discovery of new potential anti-trypanosomal compounds from natural origins is challenging due to re-isolation of the known compounds with the same reported bioactivity. To overcome this problem, a comprehensive analysis on different metabolites in complex mixtures can be achieved using several alternative methods such as “metabolite (or metabolic) fingerprinting,” “metabolite profiling” and “metabolite target analysis. In our study, the metabolomics approach was used to predict and identify potential novel bioactive components from the crude extracts leading to the rapid and high-throughput assessment of metabolites. Metabolite profiling of the active metabolites in crude extracts of natural sources is supported by dereplication in which the novel compounds from the active groups are differentiated from known compounds which have been studied previously [31]. The dereplication method is a process for screening the known metabolites from the crude extracts before further scale-up or isolation work is undertaken, to avoid repetitive work. High-resolution electrospray ionisation-liquid chromatography-mass spectrometry (HRESI-LCMS) data from both positive and negative ionisation modes were subjected to multivariate statistical analysis including unsupervised Principal Component Analysis (PCA) and Partial Least Squares-Discriminant Analysis (PLS-DA) to establish the optimal position of the discriminating plane, which would best separate classes. The high-resolution mass spectral data generated predicted molecular formulas used for dereplication of the secondary metabolites found in the crude extracts. In the final step of the metabolomics approach, the selected unique biomarkers were interpreted to putatively identify the metabolites using databases like Dictionary of Natural Products (DNP) and Antimarin in parallel to the discovery of novel bioactive natural products.

## 2. Results and Discussion

### 2.1. Dereplication Study on A. lanata Total Crude Extract

**Figure 1:**
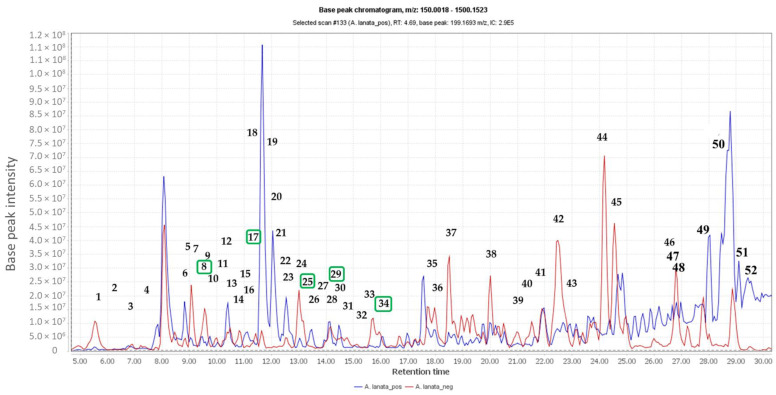
Total ion chromatogram of the crude extract of *Avicennia lanata* (blue and red lines represent positive and negative ionisation modes, respectively). Dereplication of numbered peaks is shown on Table 1. Boxed in green are the isolated compounds.

The total ion chromatogram of the crude extract of *A. lanata* (Figure 1) showed the distribution of known and unknown compounds present in the total extract (Table 1). Some of the putatively identified compounds have been previously isolated from *Avicennia* sp. (Table 1). The values and predicted formulas of unknown compounds are also shown in Table 1, indicated with “NO HITS” found. The dereplication studies revealed that the plant extract possessed certain types of compounds, such as alkaloids, triterpenes and naphthoquinones, which have also previously been isolated from different *Avicennia* species, particularly *A. marina* and *A. alba*.

The total *A. lanata* crude extract was fractionated by using medium pressure liquid chromatography with gradient elution using hexane-ethyl acetate-methanol yielding nine major fractions. To proceed with efficient targeted isolation work of the active metabolites, nine fractions were preliminarily screened against *T. b. brucei* and subjected to HRESI-LCMS prior to multivariate analysis. A dereplication study was performed to obtain the metabolomic profile of each fraction.

Each fraction was screened at different concentrations of 20, 10 and 5 μg/mL. The assay was performed in duplicate for each sample. The results show the percentage growth of *T. b. brucei* (Table 2), with negative readings representing those with higher growth inhibition on the trypanosomal cells. The *A. lanata* crude extract showed marginal anti-trypanosomal activity, whereas after fractionation, the activity for fractions F5 to F8 increased, and decreased in fraction F9. Fractions F1 and F2 showed very weak anti-trypanosomal activity whereas fractions F3, F4, and F9 showed moderate activity. Meanwhile, fractions F5 until F8 showed significant activity in this screening test, thereby supporting further investigation of the biologically active compounds from this plant extract.

**Figure 2:**
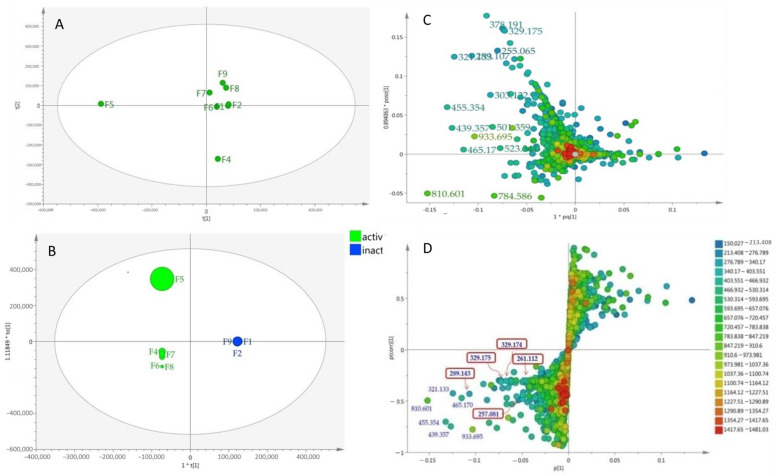
(**A**) Unsupervised Principal Component Analysis (PCA) scores plot of the *A. lanata* fractions showed moderate separation between the datasets; (**B**) OPLS-DA scores scatter plot of bioactive vs. inactive *A. lanata* fractions (R^2^(Y) = 1.00; Q^2^ = 0.998); Q^2^(Y intercept); (**C**) Supervised OPLS-DA loadings plot showed the discriminating metabolites within the fractions and (**D**) OPLS-DA S-plot of bioactive vs. inactive *A. lanata* metabolites. Boxed in red are the isolated naphthofuranquinone compounds (**1**–**5**).

The relationship between the occurrence of the metabolites in the different *A. lanata* fractions and their bioactivity against *T. b. brucei* were evaluated through multivariate analysis. The unsupervised Principal Component Analysis (PCA) scores plot showed moderate separation of the *A. lanata* fractions (Figure 2A). There was a clear separation between the bioactive fraction F5 and the other fractions that were also active against *T. b. brucei*. Fraction F4, which also possessed moderate activity, was likewise set apart from other fractions (Figure 2A). Meanwhile, a supervised multivariate OPLS-DA scores plot analysis (Figure 2B) exhibited two classes-fractions F1, F2, F3 and F9 were the inactive group and clustered very close together, while fractions F4, F5, F6, F7 and F8 were the active group, with fraction F5 being segregated from the rest of the active cluster. The segregation of F5 may indicate the presence of unique chemistry in F5. The OPLS-DA loadings plot (Figure 2C) predicted the bioactive metabolites in active fractions F4–F8 with ion peaks at *m/z* [M + H]^+^ 378.191, 329.175, 255.065, 321.133, 289.107, 303.122, 455.354, 439.357, 933.695, 465.17, 523.341, 810.601 and 784.586 as well as *m/z* [M − H]^−^ 501.359 and 933.695. These predicted anti-trypanosomally active compounds by MVA were indicated with their MZmineIDs as listed on Table 1 (highlighted rows). Peak IDs used in this table correspond to those designated for the chromatogram shown on Figure 1.

The OPLS-DA S-loadings plot (Figure 2D) exhibited the discriminating metabolites at the end points of both the active and inactive groups, respectively. From the DNP database, it was putatively determined that fractions F1 and F2 contained mostly fatty acid oils, while fractions F3 and F4 comprised of terpenoid and sterol metabolites. Metabolites from fractions F5 to F9 were putatively identified to have mainly aromatic compounds, compounds perhaps contributing towards the activity of the fractions. Some of the ion peaks in the active fractions have been dereplicated as presented in Table 1. Characteristic metabolites for the genus Avicennia were observed in fraction F5 at *m/z* [M + H]^+^ 289.143, 329.175, 329.174, 321.133, 455.35, 465.177 and 257.081 and were putatively identified as avicenol C, glycoquinone, avicennone A, ursolic acid or oleanolic acid or aegicornin, 7,9-dimethylether-(4-*O*-methyl-β-D-glucopyranoside)-3,4-dihydro-4,7,9,10-tetrahydroxy-3-methyl-1*H*-naphtho [2,3-*c*]pyran and avicequinone C, respectively; these were earlier described from *A. marina*, *A. alba* and *A. officinalis* [7,9,11]. The ion peak at *m/z* [M + H]^+^ 261.112 was dereplicated as furo[3,2-*h*][2]benzopyran-3(2*H*)-one analogues: pergillin, pseudodeflectusin and aspergione F, which were first reported from a marine-derived *Aspergillus.* The dereplication result for *m/z* [M + H]^+^ 261.112 was then later described as to be another compound after NMR analysis. Meanwhile, [M + H]^+^ at *m/z* 439.359, 261.112, 810.601 and [M − H]^−^ at *m/z* 933.695 were the unidentified metabolites found in the active fractions. 

**Figure 3:**
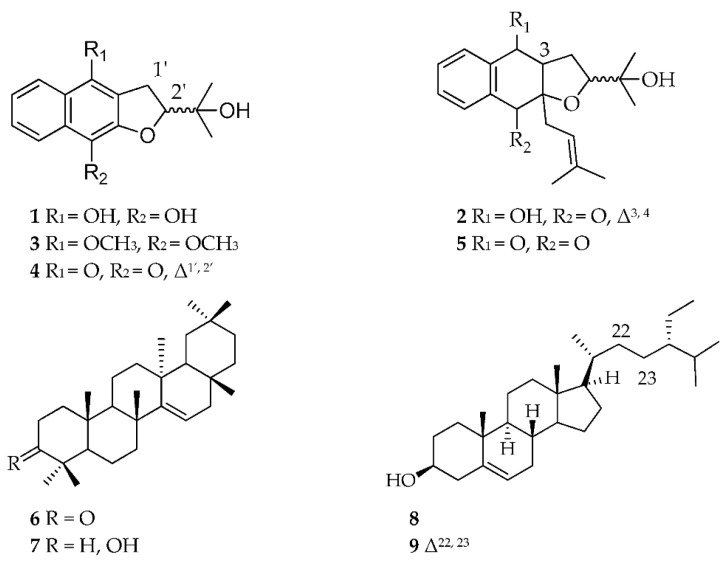
Compounds isolated from the mangrove plant *Avicennia lanata*.

**Figure 4:**
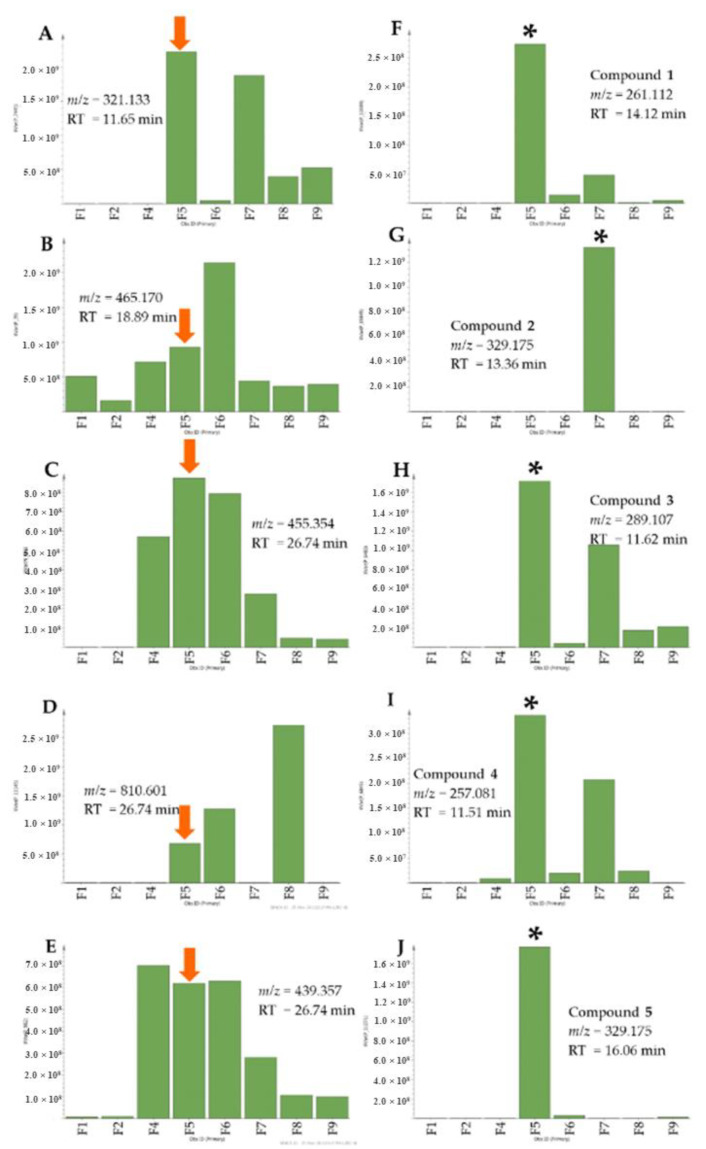
Relative abundance of target metabolites (**A**–**E**) and isolated naphthofuranquinone derivatives in the bioactive fractions (**F**–**J**). Arrows indicate the occurrence of the other target metabolites in the segregated bioactive fraction F5 as shown in Figure 2B. Asterisk specifies the fraction from where the respective compounds were isolated. (**A**) P_3575: *m/z* = 321.133, RT = 11.66 min; (**B**) P_39: *m/z* = 465.177, RT = 18.89 min; (**C**) N_243: *m/z* = 455.353; RT = 26.74 min; (**D**) *m/z* = 810.601 [M + H], RT = 29.12 min; (**E**) *m/z* = 439.357 [M + H], RT = 26.82 min; (**F**) P_3632: hydroxyavicenol (**1**), *m/z* = 261.112, RT = 14.12 min; (**G**) P_3702: semiglycoquinone (**2**), *m/z* = 329.175, RT = 13.36 min; (**H**) P_10291: avicenol C (**3**), *m/z* = 289.107, RT = 11.62 min; (**I**) P_3663: avicequinone C (**4**), *m/z* = 257.081, RT = 9.74 min and (**J**) P_3684: glycoquinone (**5**), *m/z* = 329.175, RT = 16.06 min.

The aim of this study was to isolate compounds from the active fractions that were responsible for anti-trypanosomal activity. Using metabolomics and bioassay-guided isolation work to search for new bioactive secondary metabolites against the protozoan *T. b. brucei*, the organic crude extract of *A. lanata* afforded two new metabolites, (**1**–**2**) along with the known compounds (**3**–**9**) (Figure 3) after a series of chromatographic techniques. The absolute elucidation and identification of these metabolites were achieved by using 1D and 2D-nuclear magnetic resonance (NMR) and high-resolution mass spectrometry (MS). The isolated pentacyclic triterpenes and sterols: taraxerone (**6**) taraxerol (**7**), β-sitosterol (**8**) and stigmasterol (**9**) were not initially detected from the crude extract by LCMS but were only evidenced from the NMR spectra of the extracts and the non-polar fractions, which were instead confirmed by GCMS. Meanwhile, the relative occurrence of the targeted metabolites in the bioactive fractions as well as their absence or lower abundance in the inactive fractions is shown in Figure 4.

#### 2.1.1. Compound **1** (hydroxyavicenol C)

**Figure 5:**
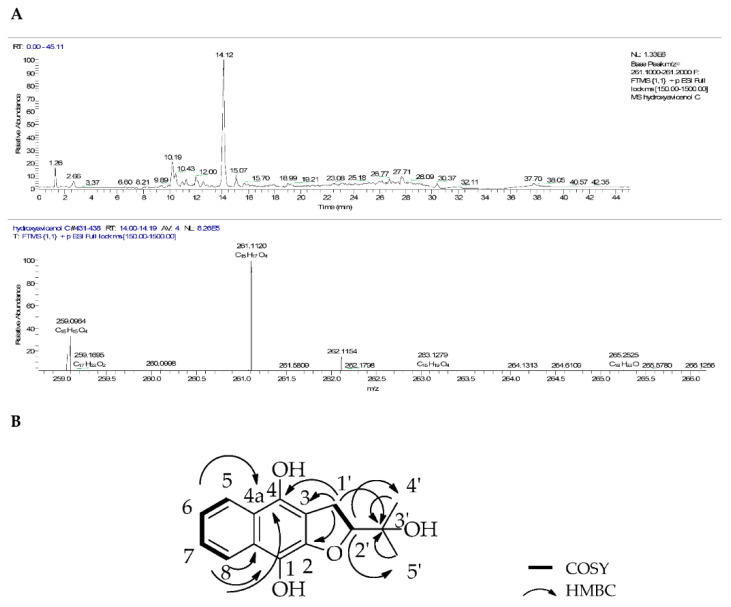
(**A**) Positive mode base peak chromatogram and mass spectrum and (**B**) COSY and HMBC correlations of compound **1**.

Compound **1** (6.5 mg) was isolated as a yellow oil and its molecular formula was determined by HRESI-MS (Figure 5A) as C_15_H_16_O_4_ with *m/z* 261.1120 [M + H]^+^ and calculated at −1.438 ppm error and optical rotation ${\left[\alpha \right]}_{D}^{20}$ −8.4° (c 1.00, CHCl_3_). The ^1^H NMR results of **1** (Table 3) showed an AB system which had two different sets of proton signals. In the aromatic region, the spectrum indicated proton signals at *δ*_H_ 8.07 (*dd*, *J* = 7.4 Hz, 2H) and 7.68 (*dt*, *J* = 7.4 Hz, 2H) corresponding to two proton units for H-5 and H-8 as well as for H-6 and H-7, respectively. In the upfield region, an oxygenated methine proton was found at *δ*_H_ 4.83 as a triplet (*J* = 10.0 Hz, 1H, H-2′). In the COSY spectrum this proton correlated to a methylene proton at *δ*_H_ 3.15 (*d*, *J* = 10.0 Hz, 2H, H-1′). Meanwhile, two proton signals were observed at 1.24 and 1.39 ppm signifying the presence of methyl groups of 4′-CH_3_ and 5′-CH_3_, respectively. The ^13^C NMR (Table 4) showed 15 carbon signals with two carbonyl carbon signals at 159.3 and 153.7 ppm, corresponding to C-4 and C-1, respectively, that were shifted downfield because of the hydroxyl substituents on both positions, while *δ*_C_ 139.3 corresponded to C-2. Four quaternary carbon signals for rings A and B were found at *δ*_C_ 129.6 (C-4a), 126.4 (C-8a) and 125.1 (C-3) as well as one quaternary carbon signal on the side chain at *δ*_C_ 71.8 (C-3′). Four aromatic carbon signals were further observed at *δ*_C_ 134.2, 133.1, 126.4 and 126.1 for C-7, C-6, C-8 and C-5, respectively. The remaining carbon signals were found at *δ*_C_ 92.4 for a methine carbon attached to oxygen (C-2′) and a methylene carbon *δ*_C_ 29.2 (C-1′) and two methyl carbon signals at 24.1 and 25.8 ppm again indicating the presence of methyl groups of 4′-CH_3_ and 5′-CH_3_, respectively.

^1^H-^1^H correlations on the COSY spectrum indicated partial structures for a tetrahydrofuran moiety, an aromatic ring and a =C-CH_2_-COH-C= unit in the molecule. Strong correlations were observed between the aliphatic doublet H-1′ and triplet H-2′ as well as between the aromatic protons. Correlations between H-1′ and C-4/C-3 in the tetrahydrofuran ring were observed in the HMBC spectrum, as well as other correlations in the aromatic AB system and tetrahydrofuran. Compound **1** was different from avicenol C (**3**), particularly on C-1 and C-4a of the naphthalene ring, where the methoxyl groups were demethylated resulting in a hydroquinone unit, while the furan ring remained the same. The other correlations between protons and carbons are depicted in Figure 5B. The optical rotation of this compound was (-), the opposite of that of **3**, implying a change of stereochemistry at position C-2′ and further suggesting compound **1** to be a new derivative. In keeping with the previously established nomenclature, the trivial name (-)-hydroxyavicenol C was proposed for (2-(2-hydroxypropan-2-yl)-2,3-dihydronaphtho-[2,3-b] furan-4, 9-diol), a *para*-hydroxyl congener of avicenol C that has not been described in the literature [9,36,37].

Meanwhile, the known naphthofuranquinone deivatives avicenol C (**3**) and avicequinone C (**4**) were also isolated in this study. The structures of the molecules were confirmed by 1D and 2D-NMR as well as comparison with previous literature, namely as avicenol C and avicequinone C, respectively.

#### 2.1.2. Compound **2** (glycosemiquinone)

**Figure 6:**
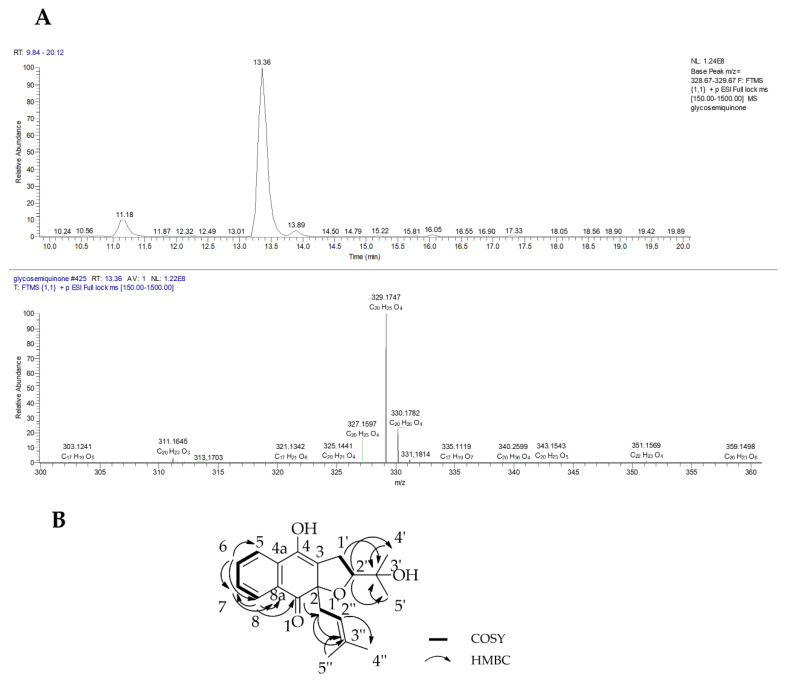
(**A**) Positive mode base peak chromatogram and mass spectrum and (**B**) COSY and HMBC correlations of compound **2**.

Compound **2** (6.0 mg) was isolated as a yellow oil and its molecular formula was determined by HRESI-MS (Figure 6A) as C_20_H_24_O_4_ with *m/z* 329.1747 [M + H]^+^, calculated at −0.504 ppm error and optical rotation ${[\alpha [}_{D}^{20}$ +11° (*c* 1.00, CHCl_3_). The ^1^H NMR results of **2** (Table **3**) showed an ABCD spin system with proton signals at *δ*_H_ 8.12 (*d*, *J* = 7.9 Hz, 1H), 7.72 (*d*, *J* = 7.8 Hz, 1H), 7.58 (*t*, *J* = 7.6 Hz, 1H) and 7.44 (*t*, *J* = 7.6 Hz, 1H) corresponding to protons H-8, H-5, H-6 and H-7, respectively. Two methyl signals located on aliphatic C-4′ and C-5′ remained the same to the known derivative glycoquinone (**5**). As in compound **5,** the presence of a prenyl moiety was indicated by one methylene proton signal at *δ*_H_ 2.98 (*m*, 2H, H-1″) that correlated to a downfield proton at *δ*_H_ 4.77 (*dd*, *J* = 8.6, 6.3 Hz, H-2″) and two proton methyl singlets at *δ*_H_ 1.49 (*s*, 3H, H-4″) and 1.54 (*s*, 3H, H-5″). Compound **2** differed to that of **5** by the incidence of a downfield methylene proton at *δ*_H_ 3.00 (*m*, 2H, H-1′) positioned on the dihydrofuran ring. The methylene proton was split to give a multiplet due to the unsaturation at carbons Δ^3,4^ and the anisotropy effect between C-3 and C-4. A methine proton signal linked to oxygen was indicated by a doublet of a doublet signal (*dd*, *J* = 9.0, 4.3 Hz, H-2′) at *δ*_H_ 3.93. 

The ^13^C NMR results (Table 4) showed 20 carbon signals, including two carbonyl carbon signals at *δ*_C_ 181.8 (C-1) and 145.6 (C-4) which was different compared with the carbon signal in compound **1**. This compound was proposed to have a carbonyl group attached at carbon position C-1. The remaining aromatic carbon signals were shown by three quaternary carbon signals at *δ*_C_ 128.9, 142.0, and 112.6, which corresponded to C-4a, C-8a and C-3, respectively. The carbon signals for prenyl moiety were observed as a methylene carbon signal at *δ*_C_ 29.6 (C-1″), olefinic carbon signals at *δ*_C_ 117.4 (C-2″) and 138.6 (C-3″) along with two methyl carbon signals at *δ*_C_ 18.7 (C-4″) and 26.3 (C-5″). The remaining aliphatic carbon signals were found similar to **5**.

The molecular structure was also established by 2D-NMR using COSY and HMBC experiments. Some of the important COSY correlations were between protons H-1′ and H-2′ and amongst the aromatic protons. The HMBC showed important C-H correlations between H-8 and the carbonyl carbon C-1 as well as methine carbons C-7 and C-8a. Further strong correlations were observed between H-1′ and C-3′, H-7 and C-8/8a, and between the prenyl proton H-2″ and C-4″/5″. The COSY and HMBC correlations were depicted in Figure 6B. The NMR spectra revealed that this compound was different only at C-4 in which the carbon carried a hydroxyl substituent hence affecting the splitting pattern of the aromatic protons when compared with compound **5** that has been described in *Glycosmis pentaphylla* [8]. Therefore, compound **2** was proposed to be a new semihydroquinone derivative of **5** and was assigned the trivial name (+)-glycosemiquinone. *Glycosmis pentaphylla* (Fam Rutaceae) is widely distributed in low altitude tropical forests of India, south China, Thailand, peninsular Malaysia, Indonesia and the Philippines Islands [38]. Interestingly, naphthofuranquinone compounds earlier reported for *Avicennia* species that included avicenol B and avicequinone C have also been isolated from the *Glycosmis pentaphylla* [8].

### 2.2. Anti-Trypanosomal and Cytotoxic Activities

The naphthofuranquinone derivatives from *A. lanata* showed promising anti-trypanosomal activity (Table 5). Among these derivatives, avicequinone C (**4**) was the most potent with an MIC of 3.12 µM. The differences in this structure compared with the other derivatives (**1–3**, and **5**) was the presence of *p*-dione with α,β-unsaturation on the core quinoid ring system resulted in increased anti-trypanocidal activity compared with the other derivatives. In contrast, the introduction of two methoxy groups to the quinoid core system of avicenol C (**3**) as opposed to the quinoid and the lack of the Δ^1′,2′^ unsaturation gave an MIC of 6.25 µM. A previous in vitro study on synthetic 2-(1-hydroxyethyl)-6-methoxynaphtho[2,3-b]furan-4,9-quinone and the natural product 2-acetyl-6-methoxynaphtho[2,3-b]furan4,9-quinone isolated from the trunk wood of *Tabebuia ochracea* [39] against different strains of the epimastigotes of *T. cruzi* showed that the compounds possessing only one methoxy group on the benzene ring exhibited the most potent activity against the protozoa with 100% growth inhibition (GI) at 13–17 μM of the tested compounds [32]. In contrast, the same study also showed that two natural products (2-acetyl-7,8-dimethoxynaphtho[2,3-b] furan-4,9-quinone and 2-(1-hydroxyethyl)-7,8-dimethoxynaphtho[2,3-b]furan-4,9-quinone) along with two synthetic compounds (5,6-dimethoxynaphtho [2,3-b]furan-4,9-quinone and 7,8-dimethoxynaphtho[2,3-b]furan-4,9-quinone), all of which possessed two methoxy groups on the benzene ring decreased the trypanocidal activity against the protozoa with GI of 45%, 22%, 45% and 25%, respectively. A study on naphthofuranquinone (−)-2,3,3-trimethyl-2-3-dihydronaphtho [2,3-b]furan-4,9-quinone, isolated from *Calceolaria sessilis,* which had three methyl groups attached to the side chain of the furan ring increased the trypanocidal activity against the epimastigotes of *T. cruzi* with 50% culture growth inhibition (GI_50_) values of 2.1–5.2 μM [33]. A furanoeremophilane derivative, maturone, which has been isolated from the roots of *Psacalium beamanii* [34], and a mixture of and its derivative, isomaturone, produced by Lewis acid catalysed Diels–Alder reaction of benzofuranquinone with piperylene, exhibited potent trypanocidal activity against *T. cruzi* [40].

However, the ability of a hydroxyl moiety to decrease the bioactivity might slightly be different to the effect of a methoxy group. The presence of the hydroxyl groups, instead of the quinoid group in (−)-hydroxyavicenol C (**1**) had decreased the trypanocidal activity with the MIC value of 12.50 µM. There was no difference in the anti-trypanosomal activity of new derivative, (+)-glycosemiquinone (**2**) and glycoquinone (**5**) as the MIC for both compounds was 12.50 µM. The new derivative (**2)** with a ketone at C-1 and a hydroxyl at C-4 showed the same activity as the parent analogue **5**. It showed that the presence of the prenyl group on both **2** and **5** decreased the activity against the protozoa. All naphthofuranquinone derivatives showed weak toxicities against normal prostate cells (PNT2A). 

Meanwhile, the pentacyclic triterpenes-taraxerone (**6**) taraxerol (**7**), and β-sitosterol (**8**) were inactive against *T. b. brucei* with MICs of 154.20, 145.30 μM and 142.30 μM, respectively (Table 5). Stigmasterol (**9**) had a very low activity with MIC values of 126.40 against the protozoa. Two pentacyclic triterpenes possessing the same taraxerane-type skeleton, **6** and **7**, have been isolated from the mangrove *A. lanata*, and differ only at C-3 where taraxerone has a carbonyl and taraxerol a β-OH. A previous study showed that β-amyrin had poor activity against bloodstream strain of *T. b. brucei* with an IC_50_ of 126.9 μM [41]. The molecular structure of β-amyrin is similar to **6**, the only difference being a shift in the double bond from Δ^14,15^ to Δ^12,13^. Taraxerol has also been isolated from the Ecuadorian plant *Cupania cinerea* and showed IC_50_ values of <10 μM against *T. b. rhodesiense* in vitro bloodstream trypomastigotes with low cytotoxicity [42]. A study on the activity of **6** and **7**, which have been isolated from the bark of *Cupania dentata* (Sapindaceae), against flagellate protozoan *Giardia lamblia* trophozoites, found that both have potential giardicidal activity with IC_50_ values of 26.7 and 37.8 μM, respectively [43]. The compounds also exhibited anti-plasmodial (*Plasmodium falciparum*), analgesic [44] and anti-inflammatory [45] activities. Other biological activities showed that **6** and **7** were also allelopathic [46] and displayed anti-fungal activity [47]. Meanwhile, the only difference between compound **8** and **9** is the presence of a double bond on the side chain at Δ^22, 23^. Compound **8** which possessed a saturated side chain was inactive against *T. b. brucei* with an MIC value of 142.30 μM. However, compound **9** which has a double bond on the side chain showed slightly increased activity compared with **8**, but still had a very low inhibitory effect on *T. b. brucei* (MIC of 126.40 μM).

## 3. Materials and Methods

### 3.1. Plant Materials

The twigs of *Avicennia lanata* were collected from Setiu Wetlands, Terengganu, Malaysia with the help of Mr Muhamad Razali Salam. The specimen was deposited in the Universiti Malaysia Terengganu herbarium with the voucher specimen code UMT-01.

### 3.2. General Experimental Procedures

The structural determination of the isolated compounds was based on MS and NMR spectroscopy data. One dimensional NMR (1D-NMR) data consisted of ^1^H and ^13^C NMR spectra captured using Jeol (^1^H 400 MHz, ^13^C 100.5 MHz, SIPBS, University of Strathclyde) and Bruker instruments (^1^H 600 MHz, ^13^C 150 MHz, Department of Pure and Applied Chemistry, University of Strathclyde) and was confirmed by two-dimensional NMR (2D-NMR) spectra such as HMQC or HSQC, HMBC, COSY and NOESY as well as comparisons with the literature. A pure sample was dissolved in 500 μL of a suitable deuterated solvent and transferred to 5 mm Norell NMR tube (NORELL Inc., Morganton, NC, USA). Samples that were low in quantity were analysed in Shigemi tubes (SHIGEMI Co., LTD., Hachioji City, Japan) with 180 μL of the appropriate deuterated solvent. Dimethyl sulfoxide-d_6_, chloroform-d, acetone-d_6,_ and methanol-d_3_ bought from Sigma-Aldrich (USA) were the deuterated solvents used. The spectra were then processed with MestReNova-9.0 (MNova) 2.10 (Mestrelab Research, S.L, Santiago de Compostela, Spain). The optical rotation of the optically active compounds was measured with the digital polarimeter 341 (PerkinElmer Life and Analytical Sciences, Shelton, CT, USA) in which the pure compound was dissolved in in 2 mL of the suitable solvent (chloroform or acetone) to a concentration of 1 mg/mL.

### 3.3. Dereplication by Using HRESI-LCMS

The dereplication study on the total crude extract and fractions of the samples were performed using HRESI-LCMS and processed with the MZmine software [48,49,50], an in-house macro coupled with the Dictionary of Natural Products (DNP) 2015 and AntiMarin 2012, a combination of Antibase and MarinLit, and SIMCA 15 (Umetrics AB, Umeå, Sweden). The procedure and program for HRESI-LCMS was set up as described below. The total crude extract of 1 mg/mL in methanol was analysed on an Accela HPLC (Thermo Fisher Scientific, Waltham, MA, USA) coupled with a UV detector at 280 and 360 nm and an Exactive-Orbitrap high-resolution mass spectrometer (Thermo Fisher Scientific, Bremen, Germany). A methanol blank was also analysed. The column attached to the HPLC was a HiChrom, ACE (Hichrom Ltd, Lutterworth, UK) C18, 75 mm × 3.0 mm, 5 μm column. The mobile phase consisted of micropore water (A) and acetonitrile (B) with 0.1 % formic acid for each solvent. The gradient program started with 10% B linearly increased to 100 % B within 30 min at a flow rate of 300 μL/min and remained isocratic for 5 min before linearly decreasing back to 10% B in 1 min. The column was then re-equilibrated with 10% B for 9 min before the next injection. The total analysis time for each sample was 45 min. The injection volume was 10 μL and the tray temperature was maintained at 12 °C. High-resolution mass spectrometry was carried out in both positive and negative ESI ionization switch modes with a spray voltage of 4.5 kV and capillary temperature at 320 °C. The mass range was set from *m/z* 150–1500 for ESI-MS range.

The mass spectral data was processed using the procedure by MacIntyre et al., [50] which was established in the Natural Products Metabolomics Group Laboratory at SIPBS as described here [35,50,51]. The LC-MS chromatograms and spectra were viewed using Thermo Xcalibur 2.1 or MZmine 2.20. 

### 3.4. Extraction, Fractionation and Isolation of Metabolites from A. Lanata 

The dried powdered twigs (4 kg) of *A. lanata* were macerated in methanol overnight (8L/extraction, 3×,) and the methanol extract was concentrated under vacuum using a rotary evaporator (Büchi Labortechnik AG, Flawil, Switzerland) to give 44.8160 g. The methanol extract was partitioned by liquid-liquid extraction three times with equal volumes of ethyl acetate to the aqueous phase (90% water + 10% methanol), to give an organic phase which was then concentrated under vacuum by a rotary evaporator (Büchi Labortechnik AG, Flawil, Switzerland) to afford the crude ethyl acetate extract weighing 14.4115 g. Thin layer chromatography (TLC) analysis was carried on *A. lanata* crude extract. The total crude extract was dissolved in any suitable solvent, mixed with Celite S (Merck KGaA, Darmstadt, Germany) then fractionated by medium pressure liquid chromatography (Büchi Labortechnik AG, Flawil, Switzerland) through gradient elution commencing with 100% hexane to 100% ethyl acetate for 20 min, followed by 100% ethyl acetate to 30% ethyl acetate and 70% methanol for 30 min at a flow rate of 50 mL/min. A VersaFlash silica column (Supelco Inc, Bellefonte, PA, USA) with dimensions of 4 × 150 mm and a particle size of 20–45 μm was used. The fraction collection volume was set at 100 mL/tube. TLC was carried out to monitor the separation profiles of the 25 fractions and similar fractions were pooled together. The pooled fractions were concentrated under vacuum by a rotary evaporator to give nine major fractions and were analysed using HRESI-MS for dereplication study and tested for anti-trypanosomal activity. The active major fractions were subjected to further isolation and purification using conventional gravity column or by Reveleris (W. R. Grace & Co.-Conn, Columbia, MD, USA) and Isolera One (Biotage AB, Uppsala, Sweden) high-throughput flash chromatography which can be either normal or reverse phase fitted with the respective commercially available pre-packed column either from Reveleris USA or SNAP Sweden, respectively. The two flash chromatography instruments were used to isolate and purify the active fractions or small quantity of the crude extracts. The non-UV active metabolites were purified by using Grace Reveleris, since dual detectors were coupled to the instrument. Meanwhile, the UV active metabolites were purified by using Biotage, since this instrument able to detect the UV active compounds in the 200–400 nm range. Open column chromatography was used with various column sizes and silica gel 60 (Kieselgel 60), 0.035–0.070 mm (220–440 mesh ASTM) (Alfa Aesar, Haverhill, MA, USA).

### 3.5. Isolation of Secondary Metabolites from A. lanata

Further purification of fraction F6 using flash chromatography (Biotage Isolera, Upsalla, Sweden and Grace Reveleris, North Chicago, IL, USA) afforded a new derivative hydroxyavicenol C (**1**, 6.5 mg, 0.05%), and further fractionation of fraction F7 by preparative thin layer chromatography resulted in one new derivative, (+)-glycosemiquinone (**2**, 6.0 mg, 0.04%). Fraction F5 was purified using open column chromatography followed by flash chromatography (Biotage Isolera, Sweden), also yielding three pure compounds: avicenol C (**3**, 20.7 mg, 0.14%), avicequinone C (**4**, 5.7 mg, 0.04%) and glycoquinone (**5**, 25.7 mg, 0.18%). Further purification of fraction F3 gave four compounds, which were taraxerone (**6**, 148.8 mg, 1.03%), taraxerol (**7**, 124.0 mg, 0.84%), 0.76%), β-sitosterol (**8**, 32.0 mg, 0.22%) and stigmasterol (**9**, 108.9 mg).

### 3.6. Bioassays

MIC assays were performed for samples having greater than 90% inhibition at a concentration of 20 μM in the initial screening campaign. The MIC values of the isolated compounds against *T. b. brucei* were determined by averaging the results of two independent assays. The concentrations were averaged and converted to μM. 

Meanwhile, the initial screening for cytotoxicity activity of the isolated compounds was performed on human normal prostatic epithelial cells (PNT2A) at 100 μg/mL. The % D of control was determined by averaging the results of three independent assays. In the initial screening, if the cell viability is less than 60%, a concentration response test at 0.3 to 300 μg/mL was carried on.

#### 3.6.1. Anti-Trypanosomal Assay

The activity of the pure compounds was tested in vitro against the blood stream form of *Trypanosomal brucei brucei* (*T. b. brucei*) S427. The activity of the samples was determined using the well-established Alamar blue™ 96-well microplate assay, in which the screening procedure was modified from the microplate Alamar blue assay [52], to determine the drug sensitivity of African trypanosomes. The samples were initially screened at one concentration (20 μg/mL crude extracts or fractions, 20 μM for pure compounds) to determine their in vitro activity. Stock solutions of tested samples in DMSO (Acros Organics BVBA, Janssen Pharmaceutical, Geel, Belgium) were prepared at concentrations of 10 mg/mL (extracts) or 10 mM for pure compounds. The concentration of DMSO should not exceed 0.5% of the final test solution. 

#### 3.6.2. Cytotoxicity Assay

The pure compounds from mangrove plant, *A. lanata* were tested for cytotoxicity in vitro against human normal prostatic epithelial cell line (PNT2A) derived from ECACC (Sigma-Aldrich, Dorset, UK). 

The cytotoxicity activity of the pure compounds was determined using the well-established Alamar Blue™ redox-based 96-well microplate assay, in which the screening procedure was modified from [53]. PNT2A cells were seeded into 96-well microplate assay at density of 0.5 × 10^4^ cells/well in 100 μL of Dulbecco’s modified Eagle’s medium (DMEM; Invitrogen, Paisley, UK) and incubated at 37 °C, 5% CO_2_ with a humidified atmosphere for 24 h. The tested compound was prepared at desired concentrations in DMEM solutions, while DMSO and Triton X as a negative and positive controls, respectively, were added into the microplate to give a total volume of 200 μL. The microplate plate was incubated at 37 °C, 5% CO_2_ with a humidified atmosphere for 24 h, then 10 μL of Alamar blue was added. The microplate was further incubated for another 20 h, then the fluorescence was measured using a Wallac Victor 2 microplate reader (Perkin Elmer, Cambridge, UK) (excitation: 530 nm, emission 590 nm). The results were calculated as % of the DMSO control values.

## 4. Conclusions

The main aims in the present work were to isolate secondary metabolites for anti-trypanosomal derived drugs from the mangrove plant, *Avicennia lanata* by utilising metabolomics and bioassays-guided approaches to aid in the preliminary screening, fractionation and purification of the targeted bioactive compounds. To achieve the goal of the discovery for new potential active secondary metabolites, the use of metabolomics tools assisted in the decision making of which fractions with targeted bioactivity should be prioritized for further isolation work. By means of high-resolution liquid chromatography-mass spectrometry, the crude fractions obtained from the *A. lanata* crude extract were preliminarily screened for bioactive molecules against *T. b. brucei* and were analysed by multivariate analysis such as PCA and OPLS-DA. A dereplication study was used to screen the known metabolites and predict the novelty of metabolites from the crude fractions prior to purification work to avoid repetitive work with the same bioactivity. Metabolomics and bioassay-guided isolation of potential anti-trypanosomal secondary metabolites were identified from the crude extracts of the mangrove plant *A. lanata*, which included two new naphthofuranquinone derivatives, hydroxyavicenol C and glycosemiquinone along with seven known compounds that included naphthoquinones, triterpenes and sterols. The naphthofuranquinone derivatives were active against the protozoa, *T. b. brucei* while the triterpenes and sterols were found inactive.

## Figures and Tables

**Table 1:** List of dereplicated putative compounds indicated on the total ion chromatogram for the crude extract of *A. lanata* that were putatively identified using DNP database. Highlighted rows represent compounds predicted to be anti-trypanosomally active by MVA as indicated with their MZmine IDs. Peak IDs used in this table correspond to those designated for the chromatogram shown on Figure 1.

Peak ID	ESI Modes/MZmineID *	Rt (min)	MS (*m/z*)	Molecular Weight	Chemical Formula	Name *	Tolerance (ppm)	Sources ^∆^	Peak Area
**1**	N_258	5.54	165.0196	166.0269	C_8_H_6_O_4_	1,4-benzenedicarboxylic acid	1.6197	*Cassia roxburghii* and *Tephrosia hamiltonii*	2.06 × 10^8^
**2**	P_7894	6.82	279.1230	278.1158	C_15_H_18_O_5_	avicennone G	1.2674	*Avicennia marina*	9.23 × 10^5^
**3**	P_3680	7.84	337.1280	336.1207	C_17_H_20_O_7_	avicennone B	−0.4533	*Avicennia marina*	5.92 × 10^7^
**4**	N_2920	8.09	261.1131	262.1203	C_15_H_18_O_4_	avicennone F	−0.605	*Avicennia marina*	9.31 × 10^5^
**5**	P_9663	9.04	261.0676	260.0604	C_15_H_13_ClO_2_	3-chlorodeoxylapachol	−0.2017	*Avicennia germinans*	3.93 × 10^5^
**6**	N_145	9.12	187.0976	188.1048	C_9_H_16_O_4_	aspinonene	−0.0654	marine-derived *Aspergillus ostianus*	2.24 × 10^8^
**7**	P_4743	9.31	215.0339	214.0266	C_12_H_6_O_4_	avicennone D or E	0.0973	*Avicennia marina*	7.07 × 10^6^
**8**	P_3663 *	9.74	257.0807	256.0735	C_15_H_12_O_4_	avicequinone C *(4)	−0.3213	*Avicennia alba*	7.90 × 10^7^
**9**	N_251	9.56	305.0668	306.0741	C_15_H_14_O_7_	fusarubin	0.525	marine-derived *Fusarium*	2.78 × 10^8^
						coniochaetone G		marine-derived *Penicillium oxalicum*	
**10**	N_3128	10.16	531.1874	532.1947	C_27_H_32_O_11_	2′-*O*-(5-phenyl-2*E*,4*E*-pentadienoyl)mussaenosidic acid	0.3802	*Avicennia marina*	5.60 × 10^5^
**11**	P_9206	10.19	245.0445	244.0372	C_13_H_8_O_5_	marinnone A or B	0.252	*Avicennia marina*	1.43 × 10^6^
**12**	N_4676	10.27	227.0715	228.0787	C_14_H_12_O_3_	avicenol B	0.4426	*Avicennia alba*	3.73 × 10^5^
**13**	P_3635	11.12	215.0339	214.0266	C_12_H_6_O_4_	avicennone D or E	−0.1166	*Avicennia marina*	1.13 × 10^8^
**14**	N_1496	11.26	389.1250	390.1323	C_20_H_22_O_8_	4′,7′,9′-trihydroxy-3,3′-dimethoxy-4,8′-oxylign-7-en-9-oic acid	2.051	*Avicennia marina*	4.29 × 10^6^
**15**	P_8572	11.40	245.0445	244.0373	C_13_H_8_O_5_	marinnone A or B	0.377	*Avicennia marina*	7.48 × 10^5^
**16**	N_920	11.42	543.1862	544.1935	C_28_H_32_O_11_	geniposidic acid	1.5797	*Avicennia marina*	2.24 × 10^6^
**17**	P_10291 *	11.62	289.1070	288.1362	C_17_H_20_O_4_	avicenol C * (3)	0.1199	*Avicennia alba* and *Avicennia officinalis*	1.15 × 10^6^
**18**	P_3575	11.66	321.1329	320.1257	C_17_H_20_O_6_	avicennone A	−1.0122	*Avicennia marina*	1.55 ×10^9^
**19**	P_4500	11.83	391.1380	390.1307	C_20_H_22_O_8_	4′,7′,9′-trihydroxy-3,3′-dimethoxy-4,8′-oxylign-7-en-9-oic acid	−1.8508	*Avicennia marina*	9.08 × 10^6^
**20**	P_4152	12.09	378.1909	377.1836	C_20_H_27_NO_6_	carpatamide D	−0.6572	marine-derived *Streptomyces*	1.48 × 10^7^
**21**	P_6066	12.10	199.0389	198.0316	C_12_H_6_O_3_	naphtho[1,2-β]furan-4,5-dione avicequinone B	−0.3764	*Avicennia marina* *Avicennia alba*	2.41 × 10^6^
**22**	P_3962	12.38	349.2007	348.1934	C_20_H_28_O_5_	(6ξ,15*S*)-6,11-epoxy-6,12,16-trihydroxy-6,7-seco-8,11,13-abietatrien-7-al	−0.8252	*Avicennia marina*	1.31 × 10^7^
**23**	P_3589	12.54	321.1332	320.0896	C_16_H_16_O_7_	6-(1-acetoxyethyl)-5-hydroxy-2,7-dimethoxy-1,4-naphthoquinone phomopsichin A or chaetochromone C rhytidchromone A	−0.1341	mangrove-derived *Botryosphaeria australis* mangrove-derived *Phomopsis* mangrove-derived *Rhytidhysteron rufulum*	2.35 × 10^8^
**24**	N_246	13.01	329.2336	330.2409	C_18_H_34_O_5_	penicitide B	0.8396	marine-derived *Penicillium chrysogenum*	2.95 × 10^8^
**25**	P_3702 *	13.36	329.1747	328.1673	C_20_H_24_O_4_	glycoquinone	−0.5072	*Glycosmis pentaphylla*	4.10 × 10^7^
**26**	P_3627	13.47	185.0597	184.0524	C_12_H_8_O_2_	4-hydroxydibenzofuran	0.1889	*Huperzia serrata*	9.19 × 10^7^
**27**	P_5879	13.70	245.1174	244.1101	C_15_H_16_O_3_	avicennone C	0.6366	*Avicennia marina*	1.90 × 10^6^
**28**	P_3603	14.04	370.1859	369.1787	C_18_H_27_NO_7_	axillarine	−0.2064	*Crotalaria* sp.	1.16 × 10^8^
**29**	P_3632 *	14.12	261.1120	260.1048	C_15_H_16_O_4_	pergillin pseudodeflectusin aspergione F	−0.2678	marine-derived *Aspergillus*	1.16 × 10^8^
**30**	P_3611	14.47	319.2267	318.2194	C_20_H_30_O_3_	conidiogenone J or K or spirograterpene A phomactin E or F or G	−0.2358	marine-derived *Penicillium* marine-derived *Phoma*	1.30 × 10^8^
**31**	P_5955	14.91	199.0390	198.0317	C_12_H_6_O_3_	naphtho[1,2-β]furan-4,5-dione avicequinone B	0.0859	*Avicennia marina* *Avicennia alba*	5.83 × 10^6^
**32**	N_256	15.72	343.2130	344.2203	C_18_H_32_O_6_	gliomasolide C or D	−0.3879	marine-derived *Gliomastix*	4.58 ×10^8^
**33**	P_4414	15.83	257.0808	256.0739	C_15_H_12_O_4_	avicequinone C	1.3625	*Avicennia alba*	6.59 × 10^5^
**34**	P_3684 *	16.06	329.1746	328.1674	C_20_H_24_O_4_	glycoquinone*(5)	−0.2747	*Glycosmis pentaphylla*	5.05 × 10^7^
**35**	N_253	17.97	311.2231	312.2303	C_18_H_32_O_4_	gliomasolide A	−0.4263	marine-derived *Gliomastix*	5.05 × 10^8^
**36**	N_241	18.51	501.3226	502.3298	C_30_H_46_O_6_	3,12,15-trihydroxy-11-oxolanosta-8,24-dien-26-oic acid	0.8476	fungus *Ganoderma applanatum*	4.69 × 10^8^
**37**	P_39	18.89	465.1775	464.1848	C_23_H_30_O_10_	7,9-dimethylether-(4-*O*-methyl-β-D-glucopyranoside)-3,4-dihydro-4,7,9,10-tetrahydroxy-3-methyl-1*H*-naphtho [2,3-*c*]pyran	1.8852	fungus *Conoideocrella luteorostrata*	5.86 × 10^5^
**38**	N_244	20.01	343.2129	344.2202	C_18_H_32_O_6_	gliomasolide C or D	0.8426	marine-derived *Gliomastix*	4.58 × 10^8^
**39**	N_273	20.98	291.1970	292.2042	C_18_H_28_O_3_	2-deoxy-5-*O*-methylembelin	1.1711	mangrove *Aegiceras corniculatum*	1.88 × 10^7^
**40**	P_5326	21.60	301.0706	300.0634	C_16_H_12_O_6_	luteolin-7-methyl ether	−0.0453	*Avicennia marina*	2.85 × 10^6^
**41**	P_3592	21.97	279.2318	278.2245	C_18_H_30_O_2_	farnesylacetone epoxide 6,10,14-trimethyl-5,10-pentadecadiene-2,12-dione monotriajaponide A	−0.2834	alga *Cystophora moniliformis* alga *Sargassum* sp. sponge *Monotria (Plakortis*) *japonica*	2.85 × 10^8^
**42**	N_240	22.48	293.2125	294.2198	C_18_H_30_O_3_	pseudopyronine B 5,8-dihydroxy-9,12-octadecadienoic acid δ lactone plakortoxide B or plakortone E 11-oxolinoleic acid 11-oxo-9,12-octadecadienoic acid	1.0809	marine-derived *Pseudomonas sp.* *Aspergillus nidulans* sponge *Plakortis simplex* alga *Lithothamnion* plant symbiont *Trichoderma*	9.57 × 10^8^
**43**	P_8752	22.82	435.2729	434.2656	C_25_H_38_O_6_	12-*O*-D-xylopyranoside-8,11,13-abietatriene-11,12-diol	−2.7234	*Avicennia marina*	2.74 × 10^6^
**44**	N_238	24.18	313.2385	314.2457	C_18_H_34_O_4_	5,8-dihydroxy-9-octadecenoic acid woodylide A, dihydroplakortin, plakortether B, 9,10-dihydro-3-epiplakortin, 3,6-epidioxy-4,6,8-triethyl dodecanoic acid	0.1418	*Aspergillus nidulans* sponge *Plakortis*	1.40 × 10^9^
**45**	N_239	24.54	295.2279	296.2352	C_18_H_32_O_3_	3-hydroxy-4,6-octadecadienoic acid 7-methoxy-9-methyl-4,8-hexa decadienoic acid	0.2707	alga *Tydemania expeditionis* blue-green alga *Lyngbya variegata* and the brown seaweed *Ishige okamurae*	1.14 × 10^9^
**46**	N_243	26.74	455.3533	456.3606	C_30_H_48_O_3_	ursolic acid or oleanolic acid aegicornin	0.6208	widely distributed in plants mangrove *Aegiceras corniculatum*	4.22 × 10^8^
**47**	N-252	26.79	933.6944	934.7016	C_59_H_86_N_10_	No hits			2.12 × 10^8^
**48**	P_3590	26.82	439.3579	438.3506	C_30_H_46_O_2_	No hits			3.55 × 10^8^
**49**	N_1704	28.04	261.1132	262.1205	C_15_H_18_O_4_	avicennone F	−0.1393	*Avicennia marina*	9.98 × 10^5^
**50**	P_3576	28.78	311.2582	310.2509	C_19_H_34_O_3_	8-hydroxy-9,12- octadecadienoic methyl ester lobophopyranone B annulofuranone 5,9-diethyl-5-hydroxy-10-tridecen-3-one acetate	0.4874	fungus *Penicillium, Aspergillus nudilans, Laetisaria arvalis* alga *Lobophora variegata* fungus *Annulohypoxylon* sponge *Plakortis*	2.58 × 10^9^
**51**	P_6110	29.10	475.3061	474.2989	C_29_H_38_N_4_O_2_	isoverbamethine; incasine C	1.2335	*Verbascum pseudonobile*	7.54 × 10^6^
**52**	P_9148	29.12	810.6008	809.6673	C_57_H_83_N_3_	No hits			8.12 × 10^5^

^∆^ As the number of “HITS” is mostly greater than 20, sources were filtered to the Genus Avicennia or biological sources only widely distributed in Southeast Asia and their marine-derived or associated endophytes that could include both bacteria and/or fungi as well as for the presence of a benzofuran unit for compounds with DBEs between 7 and 10. Albeit the number of “HITS” is less than 20 for a couple of ion peaks (e.g., P_6110), the biological source has a lower distribution to be found in the region of the collected material, then there is a higher probability that the detected metabolite could be a new natural product, which in this case cannot be properly validated. MS fragmentation interpretation was inconclusive for compounds with more than 20 HITS. For the benzofuran analogues, the neutral loss of several water units could be observed. The identification of the occurrence of respective compounds could only be validated with a reference Avicennia extract with known standard analogues in the database and by NMR after the initial fractionation. As for this instance, the dereplication result for P_3632 and P_3702 were then later described to be another compound after NMR analysis of the purified bioactive secondary metabolites. For this study, targeted isolation was only done on the predicted bioactive metabolites. *: Isolated bioactive compounds marked with * corresponding compound number used in the text.

**Table 2:** Anti-trypanosomal activity of *A. lanata* crude extract and its fractions. (calculated as mean value percentage viability) at different concentrations of 20, 10 and 5 μg/mL. Highlighted fractions showed potent anti-trypanosomal activity using a threshold of less than 50% viability of control at 5 μg/mL.

Fractions	Yield (g)	*T. b. brucei*^a^ (% D Control)		
		20 μg/mL	10 μg/mL	5 μg/mL
*A. lanata* extract	14.4115	35.0	73.7	107.1
F1	6.3254	114.0	139.1	142.1
F2	5.2059	131.0	151.6	151.4
F3	1.7074	40.6	51.7	92.8
F4	0.7330	42.9	49.0	61.3
F5	0.7130	−12.3	−10.5	3.8
F6	0.3380	0.2	−13.3	−5.1
F7	0.1343	−5.8	−4.9	48.3
F8	0.1200	−4.3	−3.8	32.8
F9	0.1570	33.3	19.6	85.8

^a^ % D, percentage viability of control (at 20 μg/mL, 10 μg/mL, 5 μg/mL) were determined by averaging of two independent assays (*n* = 2); suramin as positive control.

**Table 3:** ^1^H NMR of isolated naphthofuranquinone derivatives **1–5** (CDCl_3_, 400 MHz).

No.	^1^H NMR, *δ*_H_ (ppm, multi. *J* in Hz)				
	1	2	3	4	5
**1**	-	-	-	-	-
**2**	-	-	-	-	-
**3**	-	-	-	-	3.39 (*dd*, *J* = 9.6, 10.5 Hz, 1H)
**4**	-	-	-	-	-
**5**	8.07 (*dd*, *J* = 7.4 Hz, 1H)	7.72 (*d*, *J* = 7.8 Hz, 1H)	8.02 (*d*, *J* = 7.5 Hz, 1H)	8.18 (*m*, 1H)	8.07 (*m*, 1H)
**6**	7.68 (*dt J* = 7.4, 2.1 Hz, 1H)	7.44 (*t*, *J* = 7.6 Hz, 1H)	7.32 (br *t*, *J* = 7.6 Hz, 1H)	7.74 (*m*, 1H)	7.75 (*m*, 1H)
**7**	7.68 (*dt*, *J* = 7.4, 2.1 Hz, 1H)	7.58 (*t*, *J* = 7.6 Hz, 1H)	7.42 (br *t*, *J* = 7.5 Hz, 1H)	7.74 (*m*, 1H)	7.75 (*m*, 1H)
**8**	8.07 (*dd*, *J* = 7.4 Hz, 1H)	8.12 (*d*, *J* = 7.9 Hz, 1H)	8.00 (*d*, *J* = 7.5 Hz, 1H)	8.20 (*m*, 1H)	8.07 (*m*, 1H)
**1′**	3.15 (*d*, *J* = 10.0 Hz, 2H)	3.00 (*m*, 2H)	3.39 (*d*, *J* = 8.5 Hz, 2H)	6.81 (*s*, 1H)	2.43 (*ddd*, *J* = 4.5, 9.7, 13.7 Hz, 1H),
	-	-	-	-	1.97 (*ddd*, *J* = 9.1 10.5, 13.7 Hz, 1H)
**2′**	4.83 (*t*, *J* = 10.0 Hz, 1H)	3.93 (*dd*, *J* = 9.0, 4.3 Hz, 1H)	4.71 (*t*, *J* = 8.5 Hz, 1H)	-	3.90 (*dd*, *J* = 9.1, 4.4 Hz, 1H)
**4′-CH_3_**	1.24 (*s*, 3H)	1.21 (*s*, 3H)	1.26 (*s*, 3H)	1.70 (*s*, 3H)	1.48 (*s*, 3H)
**5′-CH_3_**	1.39 (*s*, 3H)	1.35 (*s*, 3H)	1.39 (*s*, 3H)	1.70 (*s*, 3H)	1.53 (*s*, 3H)
**6′-OCH_3_**	-	-	4.02 (*s*, 3H*)*	-	-
**7′-OCH_3_**	-	-	3.97 (*s*, 3H)	-	-
**1″**	-	2.98 (*m*, 2H)	-	-	2.54 (*m*, 2H)
**2″**	-	4.77 (*dd*, *J* = 8.6, 6.3 Hz, 1H)	-	-	5.00 (*t*, *J* = 7.7 Hz, 1H)
**4″-CH_3_**	-	1.49 (*s*, 3H)	-	-	1.48 (*s*, 3H)
**5″-CH_3_**	-	1.54 (*s*, 3H)	-	-	1.53 (*s*, 3H)
**8″-OCH_3_**	-	-	-	-	-
**9″-OCH_3_**	-	-	-	-	-

**Table 4:** ^13^C NMR of isolated naphthofuranquinone derivatives **1**–**5** (CDCl_3_, 100.5 MHz).

Atom C	^13^C NMR, *δ*c (ppm)				
	1	2	3	4	5
**1**	153.7	173.5	147.3	173.5	173.3
**2**	139.3	151.7	147.7	151.7	151.6
**3**	125.1	131.4	118.7	131.4	131.2
**4**	159.3	180.9	132.3	180.9	180.7
**4a**	129.6	132.7	129.4	132.7	132.9
**5**	126.1	126.9	121.8	126.9	126.8
**6**	133.1	133.9	125.9	133.9	133.9
**7**	134.2	133.9	123.4	133.9	133.7
**8**	126.4	127.2	121.1	127.2	126.8
**8a**	132.5	133.3	124.2	133.3	132.3
**1′**	29.2	102.7	28.8	102.7	102.6
**2′**	92.4	168.1	90.4	168.1	168.1
**3′**	71.8	69.5	71.8	69.5	69.3
**4′-CH_3_**	24.1	28.8	24.5	28.8	28.7
**5′-CH_3_**	25.8	28.8	26.2	28.8	28.7
**O-CH_3_**	-	-	60.4	-	-
**O-CH_3_**	-	-	60.7	-	-
**1″**	-	29.6	-	-	34.7
**2″**	-	117.4	-	-	116.6
**3″**	-	138.6	-	-	138.1
**4″-CH_3_**	-	18.7	-	-	18.1
**5″-CH_3_**	-	26.3	-	-	25.8
**6″**	-	-	-	-	-
**7″**	-	-	-	-	-
**8″-OCH_3_**	-	-	-	-	-
**9″-OCH_3_**	-	-	-	-	-

**Table 5:** Activities of the isolated compounds from *A. lanata* against *T. b. brucei* and PNT2A cells.

Compounds	MICs ^a^ (μM)	Cytotoxicity ^b^, % D of Control (100 μg/mL)
**1**	12.50	86.1
**2**	12.50	86.1
**3**	6.25	78.3
**4**	3.12	68.6
**5**	12.50	80.3
**6**	154.20	88.8
**7**	145.30	92.8
**8**	142.30	96.2
**9**	126.40	98.7
suramin	0.11	n.d.
triton X	n.d.	0.082

^a^ Each sample was tested in two independent assays against *T. b. brucei*, MIC values indicate the minimum inhibitory concentration of a compound/standard in μM necessary to achieve 90% growth inhibition. MICs (MIC < 10 μM—promising; 10 μM < MIC > 20 μM—moderate; 20 μM < MIC > 30 μM—marginal/weak; 30 μM < MIC > 40 μM—limited; MIC > 40 μM—no activity); ^b^ Initial screening for cytotoxicity activity against human normal prostatic epithelial cells (PNT2A), % DMSO of control values (at 100 μg/mL,) were determined by averaging of three independent assays results (*n* = 3); n.d.—not determined.

## Data Availability

The data presented in this study are available in the article.
